# The Therapeutic Aryl Hydrocarbon Receptor-Modulating Agent Tapinarof Restores Skin Barrier Dysfunction in a Mite Antigen-Induced Murine Model of Atopic Dermatitis

**DOI:** 10.3390/ijms27188032

**Published:** 2026-09-09

**Authors:** Gaku Tsuji, Masaki Takemura, Koji Kawamura, Ayako Yumine, Noriko Konishi, Takeshi Nakahara

**Affiliations:** 1 Department of Dermatology, Graduate School of Medical Sciences, Kyushu University, Fukuoka 812-8582, Japan; takemura.masaki.618@m.kyushu-u.ac.jp (M.T.); kawamura.koji.570@s.kyushu-u.ac.jp (K.K.); yumine.ayako.977@m.kyushu-u.ac.jp (A.Y.); nakahara.takeshi.930@m.kyushu-u.ac.jp (T.N.); 2 Research and Clinical Center for Yusho and Dioxin, Kyushu University Hospital, Fukuoka 812-8582, Japan; 3 Biological/Pharmacological Research Laboratories, Shionogi Takatsuki Research Center, Shionogi & Co., Ltd., Osaka 569-1125, Japan; noriko.konishi@shionogi.co.jp

**Keywords:** atopic dermatitis, tapinarof, aryl hydrocarbon receptor, skin barrier, filaggrin, loricrin

## Abstract

Atopic dermatitis (AD) is characterized by pruritus and epidermal barrier dysfunction. Tapinarof, a therapeutic aryl hydrocarbon receptor (AHR)-modulating agent, is clinically effective in AD, but its mechanisms in allergen-driven models remain unclear. We evaluated tapinarof in a Dermatophagoides farinae (Df)-induced murine model of AD, focusing on AHR-mediated barrier restoration. AD-like dermatitis was induced in female NC/Nga mice, followed by daily topical tapinarof or betamethasone for four days. Disease severity, transepidermal water loss (TEWL), histopathology, and gene/protein expression were assessed. Tapinarof significantly reduced dermatitis scores and TEWL and, unlike betamethasone, preserved epidermal architecture without suppressing ear swelling. Tapinarof upregulated the AHR-responsive genes *Cyp1a1* and *Nqo1* and induced *Ovol1*, an epidermal differentiation-associated transcription factor, with increased CYP1A1, NRF2, NQO1, and OVOL1 protein expression. Tapinarof markedly increased filaggrin (FLG)- and loricrin (LOR)-positive areas in lesional skin compared with vehicle (FLG: 11.3% vs. 3.1%; LOR: 18.9% vs. 3.9%; both *p* < 0.05), restoring FLG to a level comparable to the no-treatment control, while LOR significantly exceeded it. These findings indicate that tapinarof may restore skin barrier function in allergen-induced AD via AHR-NRF2 and AHR-OVOL1 signaling, supporting its potential as a steroid-sparing topical therapy for long-term AD management.

## 1. Introduction

Atopic dermatitis (AD) is a chronic, relapsing inflammatory skin disease characterized by eczematous lesions, pruritus, and impaired skin barrier function, affecting both children and adults worldwide [1]. Although topical corticosteroids remain the mainstay of treatment, their long-term use is associated with adverse effects such as skin atrophy, telangiectasia, and increased risk of cutaneous infections [2]. These limitations have prompted the need for safe and effective non-steroidal topical agents. For long-term control of AD, combining corticosteroids with non-steroidal topical agents is considered important. Beyond emollients, various non-steroidal topical agents have been approved as steroid-sparing options for AD, including calcineurin inhibitors, phosphodiesterase-4 (PDE4) inhibitors, and topical Janus kinase (JAK) inhibitors.

Tapinarof, a first-in-class therapeutic aryl hydrocarbon receptor (AHR) modulating agent (TAMA), has recently emerged as a promising treatment for AD [3]. Clinical trials have demonstrated its efficacy in reducing disease severity in both adult and pediatric populations. In Japanese phase 3 trials of AD patients aged 12 years and older, tapinarof cream met its primary endpoint of investigator’s global assessment (IGA) response at Week 8, and approximately 50% of patients achieved a 90% improvement in the Eczema Area and Severity Index (EASI90), with continued improvement in skin lesions observed over 52 weeks of treatment [4]. Tapinarof showed significant improvements in dermatitis and pruritus scores, along with enhanced quality of life in AD patients [4,5].

Tapinarof’s mechanism of action is mediated through activation of AHR, a ligand-activated transcription factor that responds to both endogenous and environmental ligands [6,7]. Upon ligand binding, AHR translocates into the nucleus, dimerizes with aryl hydrocarbon receptor nuclear translocator, and regulates the expression of target genes, including Cytochrome P450 family 1 subfamily A member 1 (CYP1A1) [6,7]. Nuclear factor erythroid 2-related factor 2 (NRF2) is a master transcription factor that orchestrates the cellular antioxidant response by regulating genes involved in glutathione metabolism, reactive oxygen species (ROS) detoxification, and xenobiotic clearance [8]. In keratinocytes, activation of NRF2 enhances epidermal resilience by upregulating phase II detoxifying enzymes such as NAD(P)H quinone dehydrogenase 1 (NQO1), which mitigate oxidative stress exacerbated by inflammation and environmental insults in AD [9]. Notably, AHR signaling intersects with the NRF2 pathway, forming a cooperative axis that amplifies the transcription of cytoprotective genes [8,10,11].

Beyond its antioxidative role, AHR signaling also contributes to epidermal differentiation through the regulation of ovo-like 1 (OVOL1), a zinc finger transcription factor that regulates the expression of filaggrin (FLG) and loricrin (LOR), key structural proteins of the stratum corneum [12,13]. Reduced expression of OVOL1 and its downstream targets has been observed in lesional skin of patients with AD, highlighting their relevance to barrier dysfunction [13].

Experimental evidence supports the therapeutic potential of tapinarof in murine models of AD, including those induced by MC903, a vitamin D analog [14]. Murine models induced by mite antigen such as Dermatophagoides farinae (Df), a common environmental allergen, offer a clinically relevant system that recapitulates Th2-deviated inflammation and epidermal barrier dysfunction characteristic of human AD [15]. The pharmacological efficacy of tapinarof has not been previously investigated in a Df-induced AD model.

Furthermore, it is unclear whether tapinarof can restore skin barrier integrity in AD models through activation of the AHR–NRF2 and AHR–OVOL1 axes. Therefore, we sought to evaluate the efficacy of tapinarof in a mite antigen-induced murine model of AD and to elucidate its effect on key pathways associated with skin barrier homeostasis.

## 2. Results

### 2.1. Tapinarof Attenuated Disease Severity and Improved Skin Barrier Function in a Mite Antigen-Induced Murine Model of AD

**Figure 1:**
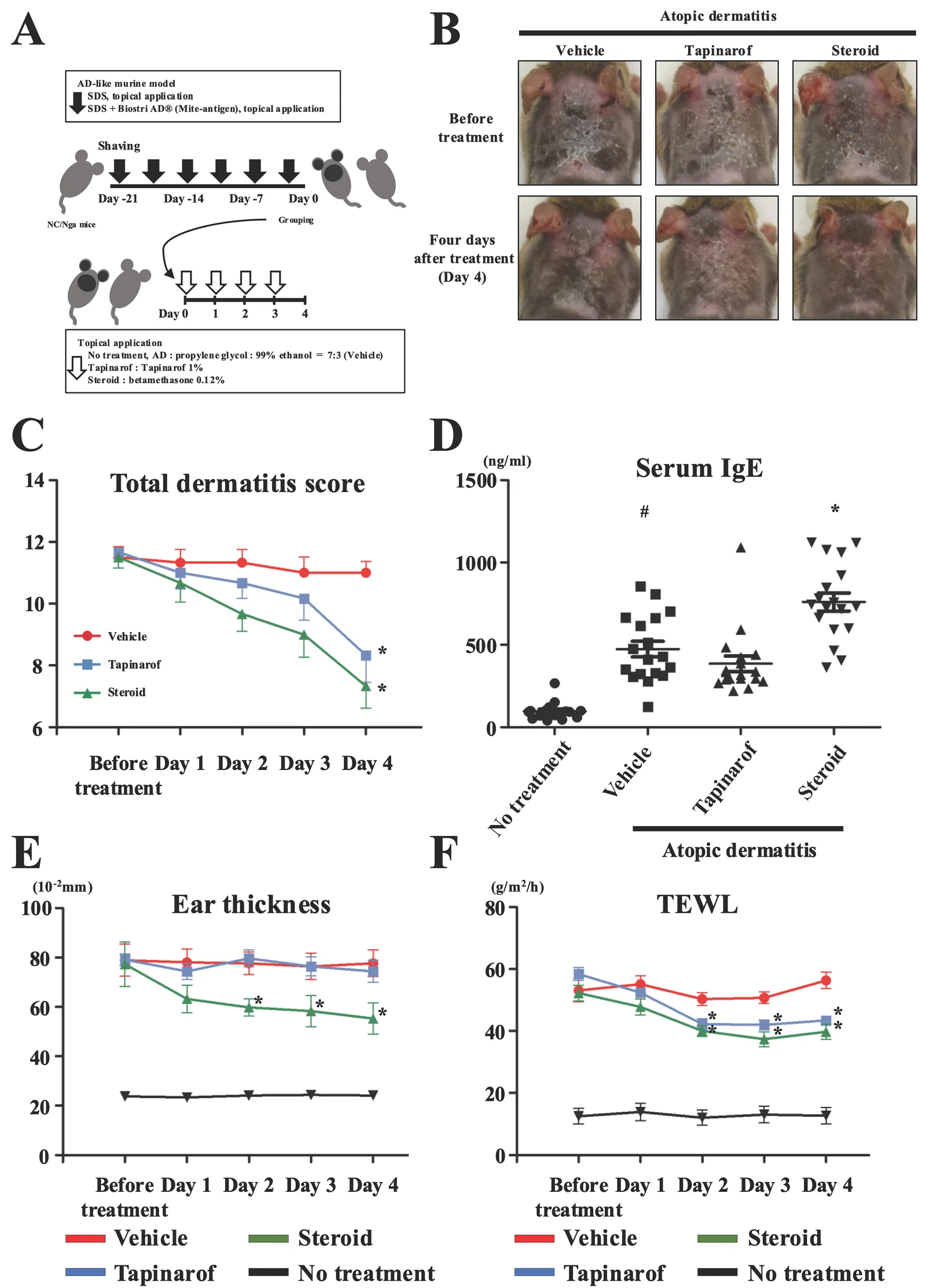
Tapinarof attenuated disease severity and improved skin barrier function in a mite antigen-induced AD murine model. (**A**) Schematic representation of the experimental protocol. Nc/Nga mice were sensitized with Dermatophagoides farinae (Df) extract twice weekly for 3 weeks. From Day 0 to Day 4, mice were treated once daily with vehicle, tapinarof (1%), or betamethasone 17-valerate (0.12%) applied topically to the dorsal skin and ears (*n* = 5 mice per group). (**B**) Representative macroscopic images of the shaved central dorsal region of the mice on Day 4. (**C**) Dermatitis scores reflecting erythema, edema, excoriation, and scaling were significantly reduced in tapinarof- and steroid-treated mice. (**D**) Serum IgE levels were significantly elevated in all three AD groups compared with the no-treatment control (all *p* < 0.05). Only the no-treatment control vs. vehicle comparison is indicated by # in the figure (# *p* < 0.05); serum IgE levels in the steroid-treated group were significantly higher than in the vehicle-treated group (* *p* < 0.05), whereas tapinarof did not differ significantly from vehicle. Data for (**D**) represent pooled results from three independent experiments (*n* = 15 per group). (**E**) Ear thickness was monitored daily; betamethasone significantly suppressed swelling by Day 2, whereas tapinarof showed no effect. (**F**) Transepidermal water loss (TEWL) measured on Day 4 was significantly reduced by both treatments, indicating improved barrier function. (**E**,**F**) Data are mean ± SEM (*n* = 5 per group), * *p* < 0.05 vehicle vs. treatment groups. Black arrows indicate the induction of atopic dermatitis, and white arrows indicate the treatment.

Repeated topical application of Df extract for 21 days was performed to induce AD-like skin inflammation. Mice were subsequently treated once daily for 4 consecutive days with vehicle, 1% tapinarof, or 0.12% betamethasone (steroid) (Figure 1A). On Day 4, visual inspection revealed notable improvement of skin lesions in both the tapinarof- and steroid-treated groups compared with that in vehicle controls (Figure 1B). Consistent with this, dermatitis scores were significantly reduced in both treatment groups (Figure 1C), indicating amelioration of local disease activity. Serum IgE levels were significantly elevated in the vehicle-, tapinarof-, and steroid-treated groups compared with those in the no-treatment control (*p* < 0.05). Serum IgE levels in the tapinarof-treated group did not differ significantly from those in the vehicle group; however, IgE levels in the steroid-treated group were significantly higher than those in the vehicle group (* *p* < 0.05) (Figure 1D). These findings indicate that, while both agents effectively suppressed local inflammation, neither treatment reduced systemic IgE-mediated responses within this treatment period, and steroid treatment was associated with a paradoxical increase in serum IgE.

To further investigate Th2-associated cytokine responses, we examined the local expression of IL-4 and IL-13 in lesional skin. Steroid treatment significantly reduced the expression of both cytokines compared with vehicle, whereas tapinarof treatment had no significant effect on either cytokine.

To assess the impact of tapinarof on inflammation and barrier integrity, we monitored ear thickness and TEWL throughout the treatment period. Steroid application significantly reduced ear thickness beginning on Day 2, indicating a rapid anti-inflammatory effect on tissue edema (Figure 1E). In contrast, tapinarof had no discernible impact on ear thickness, suggesting tapinarof may require a longer treatment duration to influence tissue edema.

In terms of barrier function, both tapinarof and steroid treatments significantly reduced TEWL by Day 4, reflecting improved stratum corneum integrity (Figure 1F). These findings suggest that tapinarof improved skin barrier function.

### 2.2. Tapinarof Induced AHR-Responsive Genes in Lesional Skin

**Figure 2:**
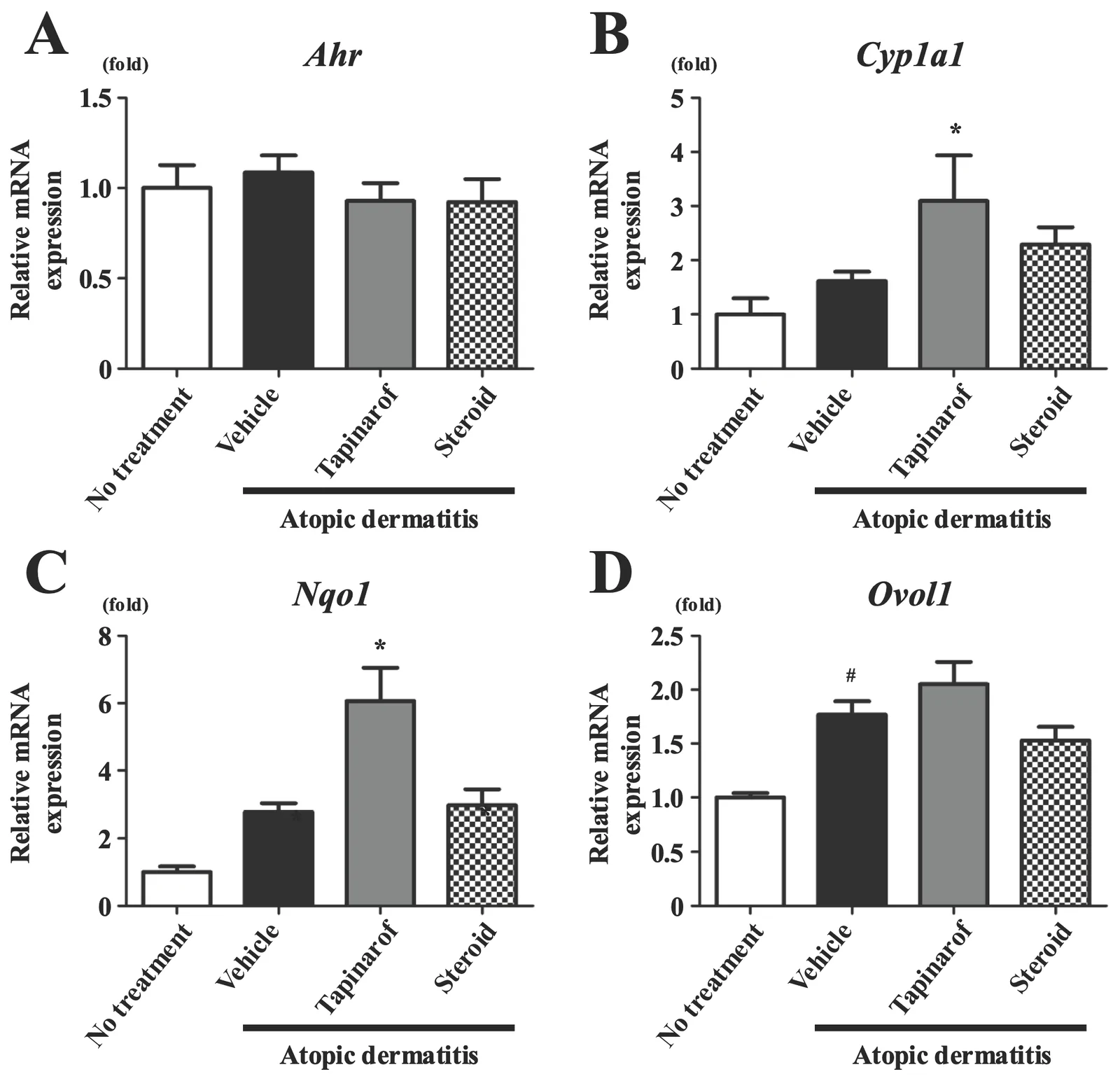
Tapinarof-induced expression of AHR-responsive genes in lesional skin. (**A**–**D**) Quantitative PCR analysis of dorsal skin tissues harvested on Day 4 after treatment with vehicle, tapinarof, or betamethasone. (**A**) Ahr, (**B**) Cyp1a1, (**C**) Nqo1, and (**D**) Ovol1 mRNA levels, normalized to β-actin. Ahr’s expression did not differ significantly among groups. Tapinarof significantly increased Cyp1a1 and Nqo1 expression compared with the vehicle, whereas steroid had no significant effect on either gene. Ovol1 expression was significantly increased in the tapinarof-treated group compared with the no-treatment control (*p* < 0.05), but not compared with the vehicle. Data are presented as mean ± SEM (*n* = 5 per group). # *p* < 0.05 no-treatment control vs. vehicle; * *p* < 0.05 vehicle vs. treatment groups.

To elucidate the molecular basis of the effects of tapinarof treatment, we quantified mRNA expression of genes associated with AHR signaling. The expression of Ahr remained unchanged across all groups, indicating that neither mite antigen stimulation nor topical treatments altered receptor transcript levels (Figure 2A).

In contrast, tapinarof significantly upregulated *Cyp1a1*, a prototypical AHR target gene, compared with vehicle; the difference between vehicle and steroid did not reach statistical significance (Figure 2B). Tapinarof also significantly upregulated Nqo1, a downstream effector of the AHR–NRF2 antioxidative pathway, compared with the vehicle, whereas steroid had no significant effect (Figure 2C). In addition, the expression of *Ovol1*, a transcription factor implicated in terminal keratinocyte differentiation, was significantly increased in the tapinarof-treated group compared with the level in the no-treatment control; this increase did not reach statistical significance when compared with vehicle, and steroid treatment had no significant effect on *Ovol1* expression relative to vehicle (Figure 2D). These results confirm in vivo activation of AHR signaling and its downstream effectors by tapinarof treatment.

### 2.3. Tapinarof Enhanced Expression of AHR-Mediated Proteins in Lesional Skin

**Figure 3:**
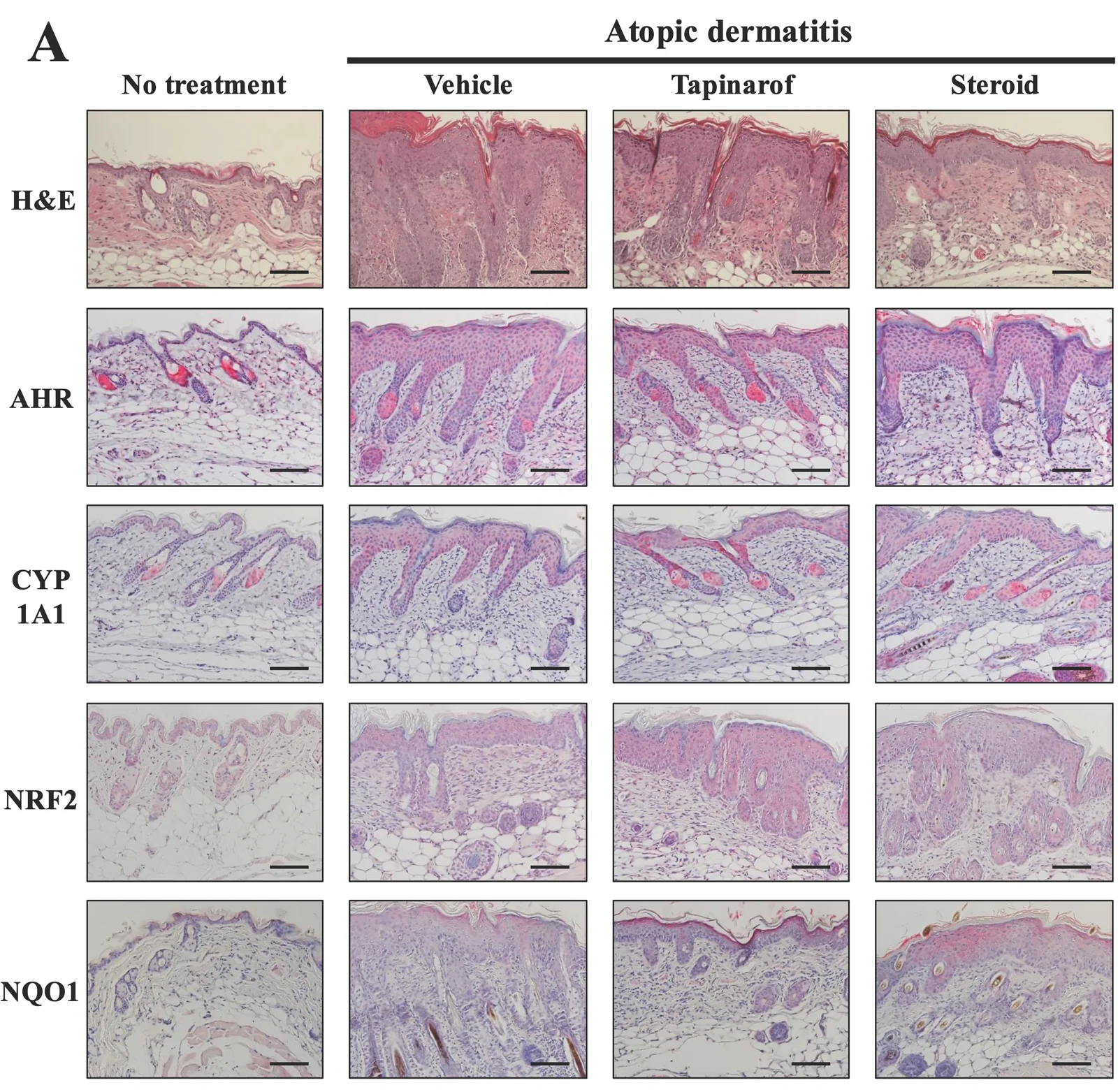

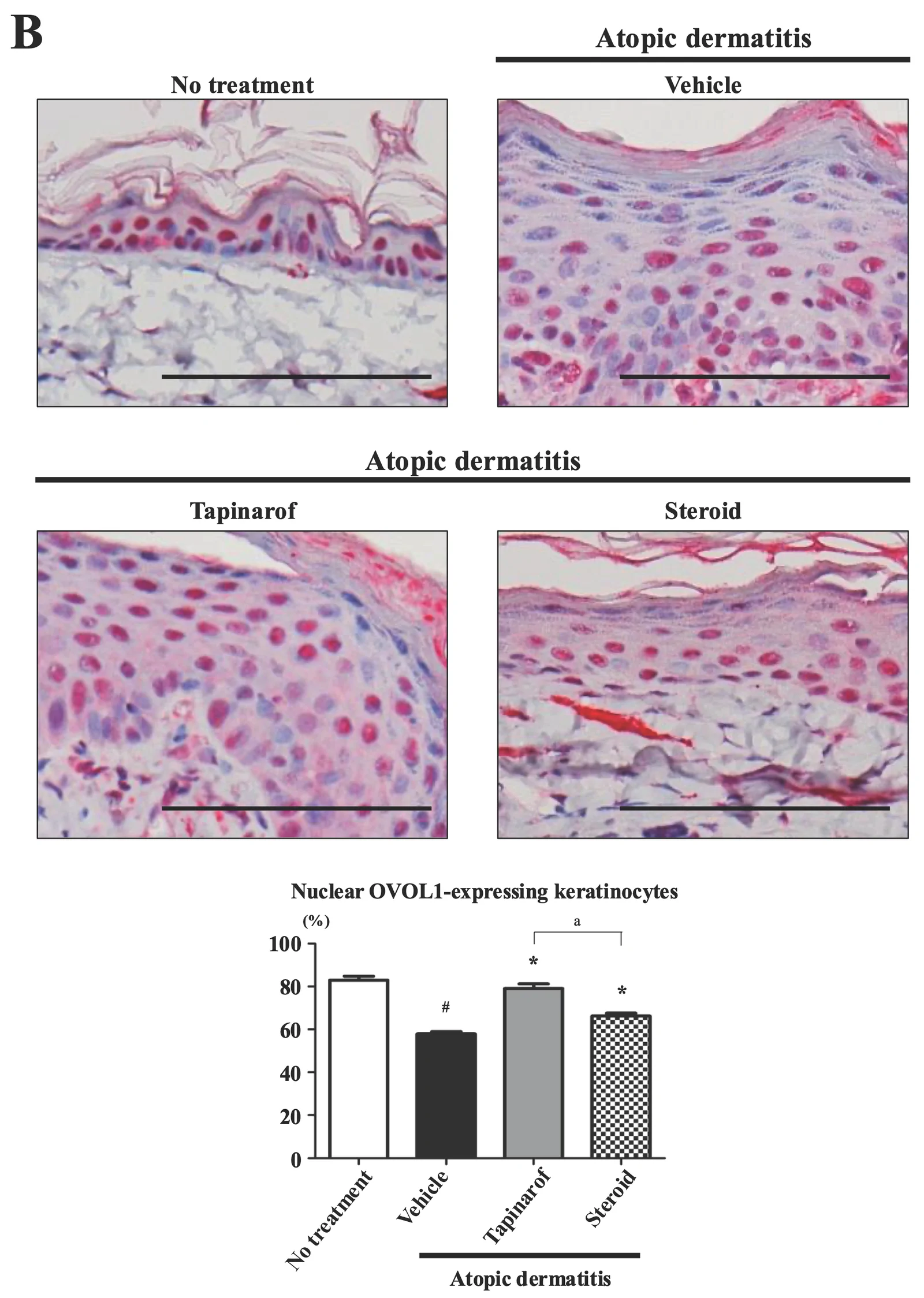
Tapinarof enhanced expression of AHR-mediated proteins in lesional skin. (**A**) H&E and immunohistochemical staining of lesional skin on Day 4. These analyses demonstrated increased expression of CYP1A1, NRF2, NQO1, and OVOL1 in the epidermis of tapinarof-treated mice compared with that in vehicle and steroid groups. CYP1A1 expression was particularly prominent in sebaceous gland openings. NQO1 was localized to the granular layer, indicating activation of antioxidative pathways. Scale bars: 50 μm. (**B**) Immunofluorescence staining showed nuclear localization of OVOL1 in keratinocytes; the proportion of nuclear Ovol1-positive keratinocytes was significantly increased in both tapinarof- and steroid-treated epidermis compared with the level with vehicle, with a more consistent increase in the tapinarof-treated group. All images are representative of at least three independent experiments. Scale bars: 20 μm. The proportion of nuclear Ovol1-expressing keratinocytes in the epidermis was calculated. # *p* < 0.05 no-treatment control vs. vehicle; * *p* < 0.05 vehicle vs. treatment groups; ^a^ *p* < 0.05 tapinarof vs. steroid.

Histological analysis using H&E staining confirmed that steroid treatment substantially suppressed epidermal hyperplasia and dermal edema, whereas tapinarof preserved epidermal structure while modestly normalizing skin morphology (Figure 3A). To validate the transcriptional changes at the protein level, we conducted immunohistochemical analysis of AHR-associated gene products. On Day 4, expression of AHR, CYP1A1, NRF2, NQO1, and OVOL1 proteins was markedly increased in the epidermis of tapinarof-treated mice relative to that in vehicle controls (Figure 3A).

CYP1A1 expression was particularly prominent at sebaceous gland openings, colocalizing with AHR and suggesting active AHR signaling in sebaceous units. NRF2 and NQO1 expression localized primarily to the granular layer of the epidermis, implicating this axis in antioxidative defense at the barrier interface. Notably, nuclear localization of OVOL1 in epidermal keratinocytes was significantly increased in both the tapinarof- and steroid-treated groups compared with that in the vehicle group, with a more consistent effect observed in the tapinarof-treated group (Figure 3B). These findings support functional activation of both the AHR–NRF2 and AHR–OVOL1 axes by tapinarof treatment in vivo, and suggest that, in the steroid-treated group, suppression of inflammation presumably resulted in partial activation of the AHR–OVOL1 axis at the protein level.

### 2.4. Tapinarof Restored FLG and LOR Expression in Lesional Skin

Given that tapinarof upregulated Ovol1 expression in AD-like skin (Figure 2D and Figure 3A,B), we next evaluated whether this effect extended to the expression of FLG and LOR. To this end, we examined their expression levels in treated skin on Day 4.

**Figure 4:**
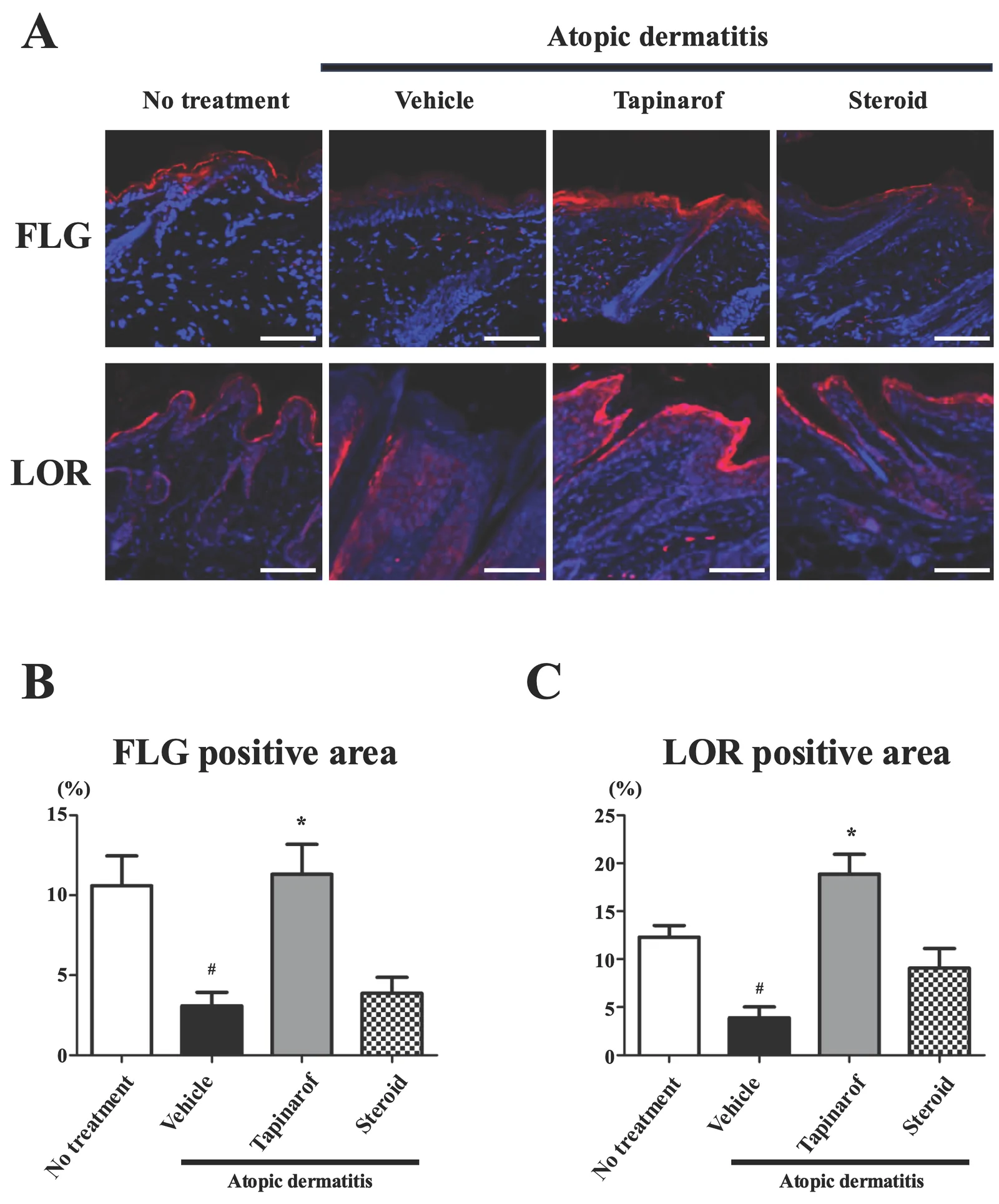
Tapinarof restored FLG and LOR expression in lesional skin. (**A**) Immunofluorescence staining of FLG and LOR in the epidermis on Day 4. (**B**,**C**) Quantification of fluorescence intensity for FLG (**B**) and LOR (**C**); data represent pooled results from three independent experiments, whereas LOR data are also shown per experiment because of a significant Group × Experiment interaction (individual experiment results in Supplementary Figure S1). Data represent mean ± SEM. Scale bars: 20 μm. # *p* < 0.05 no-treatment control vs. vehicle; * *p* < 0.05 vehicle vs. treatment groups.

Immunofluorescence staining revealed marked reductions in FLG and LOR levels in AD-like skin (Figure 4A). Tapinarof treatment restored the expression of both proteins within the epidermis, with localization primarily confined to the granular layer (Figure 4A). Quantitative analysis demonstrated that tapinarof significantly increased the fluorescence intensity of both FLG and LOR compared with vehicle, restoring FLG expression to a level not significantly different from that in the no-treatment control (Figure 4B). Steroid treatment did not significantly increase FLG expression relative to vehicle, and FLG levels in the steroid-treated group remained significantly lower than in the no-treatment control (Figure 4B). For LOR, because the effect of steroid differed significantly across the three independent experiments (Group × Experiment interaction), the results are reported for each experiment individually rather than pooled: Steroid treatment significantly increased LOR expression relative to vehicle in two of the three independent experiments, with a consistent increasing trend in the third (Figure 4C). The LOR-positive area results from each of the three independent experiments are shown in Supplementary Figure S1.

These findings suggest that the restoration of FLG and LOR expression by tapinarof is at least partially mediated via AHR–OVOL1 signaling and may underlie its superior capacity to promote epidermal differentiation and barrier repair relative to corticosteroids.

## 3. Discussion

Previous studies have demonstrated that tapinarof modulates keratinocyte differentiation and inflammation through the AHR–OVOL1–FLG axis using in vitro human keratinocyte cultures and MC903-induced murine models of AD [14]. While these findings highlighted the role of tapinarof in promoting filaggrin expression via OVOL1, they relied primarily on vitamin D3 analog-induced inflammation models, which do not fully replicate the allergen-driven Th2-skewed immune responses characteristic of human AD. In contrast, our study employed a mite antigen-induced murine model that more closely mimics the clinical pathophysiology of AD. We demonstrated that topical tapinarof application ameliorates dermatitis severity and restores skin barrier function, as evidenced by reduced dermatitis scores and TEWL.

Although both tapinarof and corticosteroid treatment improved clinical severity and barrier function, their underlying mechanisms appeared distinct. Steroid application significantly reduced ear thickness and alleviated dermal edema and epidermal hyperplasia histologically, consistent with its known anti-inflammatory and vasoconstrictive effects [16]. In contrast, tapinarof did not reduce ear swelling but showed milder histological changes (Figure 3A), suggesting that it may exert its effects without inducing dermal atrophy—an important consideration given the long-term safety concerns associated with corticosteroids.

Mechanistically, we showed that tapinarof activated cutaneous AHR signaling in vivo, as evidenced by the upregulation of *Cyp1a1*, a canonical AHR target gene. Immunostaining also revealed that AHR and CYP1A1 expression was strongly induced in sebaceous glands following tapinarof treatment. This aligns with previous reports showing that leucine-rich repeat- and immunoglobulin-like domain 1-positive sebocyte progenitors robustly induce CYP1A1 in response to an AHR ligand [17]. These observations suggest that sebaceous units may contribute to AHR-mediated responses and local drug metabolism in the skin. Furthermore, tapinarof increased the expression of Nqo1, a downstream gene of the AHR–NRF2 axis, indicating enhanced antioxidant capacity in lesional skin. Immunofluorescence staining revealed that NRF2 and NQO1 expression was prominent in the granular layer, where keratinocytes face oxidative stress from environmental insults. These results reinforce the notion that AHR–NRF2 signaling plays a key role in preserving epidermal homeostasis in AD.

In addition to antioxidant defenses, we found that tapinarof upregulated *Ovol1* expression in lesional skin. OVOL1 facilitates epidermal differentiation and supports the expression of key barrier proteins such as FLG and LOR. Tapinarof restored FLG and LOR protein levels in the granular layer, which are decreased in this AD-like model. Notably, although steroid treatment also significantly increased nuclear OVOL1 localization relative to vehicle (Figure 3B), this increase was less consistent than that observed with tapinarof, and, unlike tapinarof, steroid treatment did not restore FLG expression to a level comparable to that in the no-treatment control (Figure 4B). These findings suggest that tapinarof enhances skin barrier repair through activation of the AHR–OVOL1 axis, providing mechanistic insight into its barrier-restorative properties.

Importantly, we demonstrated *in vivo* activation of AHR-mediated transcriptional programs not only by assessing mRNA levels of canonical target genes such as *Cyp1a1* and *Nqo1*, but also by performing immunohistochemical staining to localize AHR-associated proteins within the skin. We specifically visualized the nuclear localization of OVOL1 in epidermal keratinocytes following tapinarof treatment, a finding that strongly supports its function as a transcription factor *in vivo*.

Previously, mRNA levels of *Flg* and *Lor* were measured in the MC903-induced AD murine model; however, their protein expression and localization were not evaluated. In our study, immunofluorescence staining clearly demonstrated restoration of FLG and LOR protein expression in the granular layer of AD-like skin. Collectively, these findings emphasize the mechanistic distinction and translational relevance of our study, highlighting the ability of tapinarof treatment to restore skin barrier function by modulating both epidermal differentiation and antioxidative defense in a clinically relevant AD model.

Although steroid treatment significantly reduced local IL-4 and IL-13 expression, tapinarof did not significantly alter the level of either cytokine. The local anti-inflammatory effect of steroid was not accompanied by reduced serum IgE; indeed, IgE levels were significantly higher in the steroid-treated group than in the vehicle-treated group (Figure 1D), possibly reflecting the long half-life of IgE produced by long-lived plasma cells. These findings indicate that local anti-inflammatory efficacy does not necessarily translate into reduced systemic Th2-mediated humoral responses within this timeframe, and that tapinarof and steroids may act through distinct local versus systemic mechanisms.

The beneficial effects of tapinarof observed in this study, together with their statistically significant differences relative to vehicle, are summarized in Table 1 alongside the corresponding effects of steroid treatment.

Although these findings advance the understanding of the differential actions of tapinarof in comparison with corticosteroids, this study has certain limitations. First, our experimental protocol employed a short-term treatment model, which may not fully reflect the chronicity and fluctuating nature of human AD. Long-term studies are warranted to evaluate the sustained efficacy and safety of tapinarof under chronic exposure conditions. In addition, functional validation using AHR- or OVOL1-deficient mice would be necessary to definitively establish the causal roles of these pathways in mediating the therapeutic effects of tapinarof treatment.

Second, we did not directly inhibit AHR in vivo to definitively establish that the tapinarof effects are solely due to AHR activation. The induction of *Cyp1a1* expression, a canonical AHR target, observed in our model following tapinarof treatment provides supporting evidence that effects following tapinarof therapy likely result from AHR agonism. This is further supported by data from our previous in vitro studies on primary human keratinocytes, which demonstrated that AHR knockdown abrogated tapinarof-mediated induction of AHR-responsive genes [3,18]. These findings indirectly support AHR involvement and strengthen the interpretation of our in vivo results.

## 4. Materials and Methods

### 4.1. Animals

Female Nc/Nga mice (6–10 weeks old) were obtained from Japan SLC Inc. (Shizuoka, Japan). The mice were housed under specific pathogen-free conditions at a controlled temperature (20–26 °C), humidity (40–70%), and a 12 h light/dark cycle (lights on from 08:00 to 20:00). All procedures were approved by the Institutional Animal Care and Use Committee of Kyushu University (Protocol number: A25-242-1, Approval Date: 10 November 2025–31 March 2028). Upon arrival, mice were quarantined and observed for general health status during a 2-week acclimatisation period before the start of experimental procedures; all animals were newly purchased for this study and had not been used in any prior procedures. Mice were group-housed (5 per cage) and fed a standard diet (CRF-1LID10); no environmental enrichment was provided.

### 4.2. Induction of Atopic Dermatitis

AD-like skin inflammation was induced using Df extract (Biostir^®^ AD; Biostir Inc., Osaka, Japan), based on a previously described protocol [19]. The dorsal hair of 7-week-old Nc/Nga mice was shaved, and 150 μL of 4% SDS (Nacalai Tesque, Kyoto, Japan) was applied to the skin and allowed to air-dry for 3 h. Subsequently, 100 mg of Biostir^®^ AD was applied to the shaved dorsal skin and bilateral ears using a spatula. This sensitization protocol was repeated twice weekly for either 2 or 3 weeks.

### 4.3. Topical Drug Application

Tapinarof (1%) and betamethasone 17-valerate (0.12%) were prepared using a vehicle composed of 70% propylene glycol and 30% ethanol. Tapinarof was kindly provided by Shionogi & Co., Ltd. Betamethasone and propylene glycol were purchased from Sigma-Aldrich (St. Louis, MO, USA), and ethanol was from Nacalai Tesque. Topical application of tapinarof or betamethasone was initiated on Day 0 and continued once daily for 4 consecutive days. The treatment area included the shaved dorsal skin (2 × 2 to 2 × 3 cm) and both ears. A total volume of 150 μL (100 μL to the back and 50 μL to the ears) was applied per mouse per treatment. Control and untreated AD groups received vehicle alone. After confirmation of AD-like sensitization, mice were randomly allocated to treatment groups using a random-number generator to balance baseline dermatitis severity across groups; cage position was rotated during the treatment period to minimise positional bias.

### 4.4. Clinical Scoring and Measurement

The severity of dermatitis was assessed by scoring erythema/hemorrhage, scarring/dryness, edema, and excoriation/erosion on a scale of 0 to 3, based on a previously described scoring system [19]. Ear thickness was measured using a dial thickness gauge (G-1A, Ozaki Mfg. Co., Ltd., Tokyo, Japan). Transepidermal water loss (TEWL) was measured using a Tewameter™ HEX (Courage + Khazaka, Cologne, Germany) with an MPA6 adapter. Measurements were performed on the center of the shaved dorsal skin under anesthesia, avoiding visibly damaged areas. Dermatitis scoring, TEWL measurement, and quantification of immunostaining and qPCR data were performed by an assessor blinded to treatment allocation. Mice were monitored for pre-defined humane endpoints, including skin ulceration/erosion impairing normal behaviour, long-term abnormal appearance with no expected recovery, rapid body-weight loss (≥20% within one week), or self-injurious behaviour; body weight was measured at each treatment application and weekly. Mice reaching any endpoint would have been euthanized immediately by sevoflurane overdose. No unexpected adverse events occurred, and no animals reached a humane endpoint during the study.

### 4.5. Anesthesia

All experimental procedures involving skin preparation (hair removal, SDS application, Biostir^®^ AD application), topical drug application, and blood collection were performed under sevoflurane inhalation anesthesia (induction concentration: 5%; maintenance concentration: 2.5–4%) using a small-animal anesthesia apparatus.

### 4.6. Histological Analysis

Skin tissues were collected from the shaved central dorsal region of the mice, fixed in 10% formalin, embedded in paraffin, and sectioned at 3 μm for H&E and immunohistochemical staining. Frozen sections embedded in OCT compound were used for immunofluorescence. Target antigens included AHR, CYP1A1, NRF2, NQO1, OVOL1, FLG, and LOR. Immunostaining protocols included deparaffinization, antigen retrieval (when required), blocking, incubation with primary and secondary antibodies, and counterstaining with hematoxylin or DAPI.

### 4.7. RNA Isolation and Quantitative PCR

Total RNA was extracted from dorsal skin tissues using TRIzol Reagent (Thermo Fisher Scientific, Waltham, MA, USA) and the RNeasy^®^ Mini Kit (QIAGEN, Hilden, Germany). Reverse transcription was performed using PrimeScript™ RT Reagent Kit (Takara Bio Inc., Kusatsu, Shiga, Japan), and qRT-PCR was performed using TB Green^®^ Premix Ex Taq on a CFX Connect™ Real-Time PCR System (Bio-Rad Laboratories, Inc., Hercules, CA, USA). Expression levels were normalized to β-actin. Primer sequences for *Ahr*, *Cyp1a1*, *Nqo1*, *Ovol1*, *Flg*, *Lor*, and *β-actin* are listed in Supplementary Table S1.

### 4.8. Immunofluorescence Staining

Paraffin-embedded and frozen skin sections were stained using specific antibodies against AHR, CYP1A1, NRF2, NQO1, OVOL1, FLG, and LOR. Sections were processed in accordance with standard protocols, including fixation, permeabilization, blocking, and staining with primary and fluorescence-conjugated secondary antibodies. DAPI was used for nuclear counterstaining. Stained sections were analyzed by fluorescence microscopy.

### 4.9. Serum IgE Measurement

Blood samples were collected via cardiac puncture under anesthesia. Serum was separated by centrifugation and stored at −80 °C. Total serum IgE levels were quantified using a mouse IgE ELISA kit (Thermo Fisher Scientific), in accordance with the manufacturer’s instructions.

### 4.10. Statistical Analysis

Data were analyzed using GraphPad Prism version 5.02 (GraphPad Software, Inc., San Diego, CA, USA). For Figure 1 (dermatitis score, serum IgE, ear thickness, and TEWL), Figure 2 (Ahr, Cyp1a1, Nqo1, and Ovol1 mRNA expression), and Figure 3B (nuclear OVOL1-positive keratinocyte proportion), differences among the no-treatment control, vehicle, tapinarof, and steroid groups were assessed by one-way ANOVA followed by Dunnett’s multiple comparisons test. Cyp1a1 mRNA expression data (Figure 2B) were log-transformed prior to analysis to satisfy the assumptions of normality.

For Figure 4B (FLG) and Figure 4C (LOR), quantitative data from three independent experiments were first evaluated for poolability using a two-way ANOVA (Group × Experiment) interaction test. When the interaction was not significant, percentage data were arcsine-square-root-transformed, and the pooled data were analyzed by one-way ANOVA followed by Dunnett’s multiple comparisons test, as for Figure 1, Figure 2 and Figure 3; this approach was applied to the FLG data (Figure 4B) and to the tapinarof-versus-control comparisons for LOR (Figure 4C). For the steroid-versus-vehicle comparison of LOR expression, the Group × Experiment interaction was significant; these data are therefore presented for each of the three independent experiments individually (Figure 4C; Supplementary Figure S1) rather than as pooled values. *p*-values less than 0.05 were considered statistically significant.

Generative AI was not used in any portion of the manuscript writing, data analysis, or figure generation.

## 5. Conclusions

In summary, our findings reveal that tapinarof may improve skin barrier function in mite antigen-induced AD by activating distinct AHR-mediated pathways, particularly those involving NRF2 and OVOL1. This suggests that tapinarof may offer a steroid-sparing strategy for the long-term management of AD, emphasizing barrier restoration as a key therapeutic target.

## Figures and Tables

**Table 1:** Summary of the effects of tapinarof and steroid treatment relative to vehicle in this study. In Df-sensitized mice with atopic dermatitis, ↑ indicates a significant increase and ↓ indicates a significant decrease compared with the level in the vehicle-treated (AD) group; N.C. indicates no significant change compared with the vehicle group.

Parameter	Tapinarof	Steroid
Dermatitis score	↓	↓
TEWL	↓	↓
Ear thickness	N.C.	↓
Serum IgE	N.C.	↑
Cyp1a1 mRNA	↑	N.C.
Nqo1 mRNA	↑	N.C.
Ovol1 mRNA	N.C. ^a^	N.C.
Nuclear OVOL1 (protein)	↑	↑ ^b^
FLG (protein)	↑ ^c^	N.C.
LOR (protein)	↑ ^d^	↑ ^e^

^a^ Significantly increased vs. no-treatment control, but not significantly different from vehicle. ^b^ Significant increase was less consistent than with tapinarof. ^c^ Restored FLG expression to a level comparable to that in the no-treatment control. ^d^ LOR expression significantly exceeded the level in the no-treatment control. ^e^ Significant in two of the three independent experiments; not pooled due to a significant Group × Experiment interaction.

## Data Availability

The datasets generated during the current study are available from the corresponding author on reasonable request.

## Supplementary Materials

The following supporting information can be downloaded at https://www.mdpi.com/article/10.3390/ijms27188032/s1.

## Abbreviations

The following abbreviations are used in this manuscript:

AD	Atopic dermatitis
AHR	Aryl hydrocarbon receptor
TAMA	Therapeutic AHR-modulating agent
NRF2	Nuclear factor erythroid 2-related factor 2
NQO1	NAD(P)H quinone dehydrogenase 1
OVOL1	Ovo-like 1
FLG	Filaggrin
LOR	Loricrin
TEWL	Transepidermal water loss
Df	Dermatophagoides farinae
qRT-PCR	Quantitative reverse transcription polymerase chain reaction
ELISA	Enzyme-linked immunosorbent assay
