# Supplementary material for: The Therapeutic Aryl Hydrocarbon Receptor-Modulating Agent Tapinarof Restores Skin Barrier Dysfunction in a Mite Antigen-Induced Murine Model of Atopic Dermatitis
Source: Int J Mol Sci. 2026 Sep 9;27(18):8032. doi: 10.3390/ijms27188032 (PMC13606631; doi:10.3390/ijms27188032)
Supplement: Supplementary file 1 [file ijms-27-08032-s001.zip › Supplementary Figure S1.pdf]

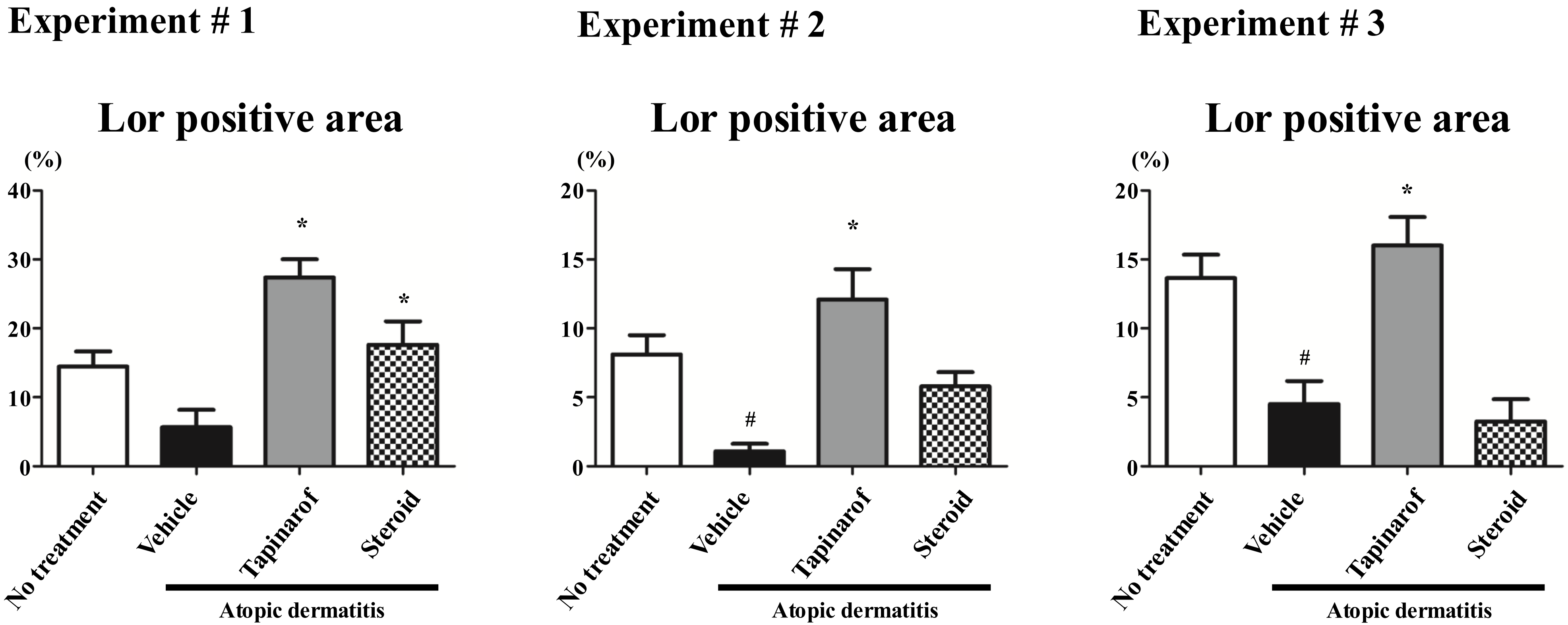

Figure S1. Immunofluorescence staining of LOR in the epidermis on Day 4. Quantification of fluorescence intensity for LOR are shown per experiment because of a significant Group × Experiment. Data represent mean ± SEM. # $p < 0.05$ vs. no treatment control; * $p < 0.05$ vs. vehicle.
