# Supplementary material for: The Therapeutic Aryl Hydrocarbon Receptor-Modulating Agent Tapinarof Restores Skin Barrier Dysfunction in a Mite Antigen-Induced Murine Model of Atopic Dermatitis
Source: Int J Mol Sci. 2026 Sep 9;27(18):8032. doi: 10.3390/ijms27188032 (PMC13606631; doi:10.3390/ijms27188032)
Supplement: Supplementary file 1 [file ijms-27-08032-s001.zip › Supplementary Table S1.pdf]

Table S1. Primer sequences.

		Sequence (5' to 3')
β-actin	forward	GGCTGTATTCCCCTCCATCG
	reverse	CCAGTTGGTAACAATGCCATGT
Ahr	forward	GCCCTTCCCGCAAGATGTTAT
	reverse	TCAGCAGGGGTGGACTTTAAT
Cyp1a1	forward	TCCTCCGTTACCTGCCTAACTC
	reverse	GATGTGGCCCTTCTCAAATGTCC
Nqo1	forward	AGCTTTAGGGTCGTCTTGGC
	reverse	ACAATCAGGGCTCTTCTCGC
Ovol1	forward	GTCACAACGACGTCAAGAGG
	reverse	CTCACACACGTAGAGCTTGG
Flg	forward	TGAAGACTGGGAGGCAAGCTA
	reverse	GACTGGGGTTCCTTCTGGGA
Lor	forward	CCCTGGTGCTTCAGGGTAAC
	reverse	CTTTCCACAACCCACAGGAGG
